# Supplementary material for: Maternal COVID-19 Serological Changes—Comparison between Seroconversion Rate in First and Third Trimesters of Pregnancy and Subsequent Obstetric Complications: A Cohort Study
Source: Viruses. 2023 Dec 5;15(12):2386. doi: 10.3390/v15122386 (PMC10747315; doi:10.3390/v15122386)
Supplement: Supplementary file 1 [file viruses-15-02386-s001.zip › viruses-2711391-supplementary.pdf]

Table S1. Serological and clinical characteristics of the 48 women with positive COVID third-trimester serology with prior negative testing in first trimester of pregnancy.

INTERPRETATION 1T	NO SYMPTOMS	MILD SYMPTOMS	SEVERE SYMPTOMS	IgA 3T	IgG 3T	rRT-PCR	POSITIVE ADDITIONAL TEST	INTERPRETATION 3T
Negative	X			+	-			EXPOSURE
Negative	X			+	+/-			EXPOSURE
Negative	X			+	-			EXPOSURE
Negative	X			+	-			EXPOSURE
Negative	X			+	-			EXPOSURE
Negative	X			+	-			EXPOSURE
Negative	X			+	-			EXPOSURE
Negative	X			+	+/-			EXPOSURE
Negative	X			+	-			EXPOSURE
Negative	X			+	-			EXPOSURE
Negative	X			+	-			EXPOSURE
Negative	X			+	-			EXPOSURE
Negative	X			+	-			EXPOSURE
Negative	X			+	-			EXPOSURE
Negative	X			+	-			EXPOSURE
Negative	X			+	-			EXPOSURE
Negative	X			+	-			EXPOSURE
Negative	X			+	-			EXPOSURE
Negative	X			+	-			EXPOSURE
Negative	X			+	-			EXPOSURE
Negative	X			+				EXPOSURE
Negative	X			+	-			EXPOSURE
Negative	X			+	-			EXPOSURE
Negative	X			+	+		X (SEM 39)	RECENT INFECTION
Negative	X			+	+			PAST INFECTION
Negative	X			+	+			PAST INFECTION
Negative	X			-	+			PAST INFECTION
Negative		X		+/-	+		X	PAST INFECTION
Negative	X			-	+			PAST INFECTION
Negative		X		+	-		X	PAST INFECTION
Negative	X			+	+			PAST INFECTION
Negative			X	+	+			PAST INFECTION
Negative		X		+	+			PAST INFECTION
Negative		X		+	+		X	PAST INFECTION
Negative		X		+	+			PAST INFECTION
Negative		X		+/-	+/-		X	PAST INFECTION
Negative	X			-	+			PAST INFECTION
Negative		X		+	+/-			PAST INFECTION
Negative		X		+	+		X	PAST INFECTION
Negative	X			+	+			PAST INFECTION
Negative		X		+	-			PAST INFECTION
Negative	X			+	+			PAST INFECTION
Negative		X		+	+	+		PAST INFECTION
Negative	X			+	+			PAST INFECTION
Negative	X			+	+			PAST INFECTION
Negative		X		-/+	x		X	PAST INFECTION
Negative		X		+	+			PAST INFECTION
Negative		X		+	+			PAST INFECTION

Classification was performed as follows: presence of IgG anti-SARS-CoV2 was classified as “past infection”; isolated IgA with no COVID-19-related symptoms was considered as “exposure to viral infection”; and isolated IgA and either medical history of COVID-19-related symptoms, positive contact prior to blood extraction, or a positive result for an additional SARS-CoV2 test were considered “recent infection”;

Table S2. Pregnant women with persistent COVID-19 serology in the third trimester of pregnancy.

IgA 1T	IgG 1T	POSITIVE CONTACT	NO SYMPTOMS	MILD SYMPTOMS	SEVERE SYMPTOMS	INTERPRETATION 1T	IgA 3T	IgG 3T	POSITIVE rRT-PCR ADDITIONAL TEST	INTERPRETATION 3T
+	-		X			EXPOSURE	+	-		EXPOSURE
+	-		X			EXPOSURE	+	-	X	EXPOSURE
+	-		X			EXPOSURE	+	-		EXPOSURE
+	-		X			EXPOSURE	+	-		EXPOSURE
+	-		X			EXPOSURE	+	-		EXPOSURE
+	-			X		RECENT INFECTION	+	-		PAST INFECTION
+	-			X		RECENT INFECTION	+	-		PAST INFECTION
+	+		X			PAST INFECTION	+	+		PAST INFECTION
+	+			X		PAST INFECTION	+	+		PAST INFECTION
+	+			X		PAST INFECTION	+	+	X	PAST INFECTION
+	+			X		PAST INFECTION	+	+	X	PAST INFECTION
+	+			X		PAST INFECTION	+/-	+/-		PAST INFECTION
+	+	X		X		PAST INFECTION	+	+	X	PAST INFECTION
+	+			X		PAST INFECTION	+	+	X	PAST INFECTION
+	+		X			PAST INFECTION	+	+		PAST INFECTION
+	+		X			PAST INFECTION	+	+	X	PAST INFECTION
+	+		X			PAST INFECTION	+	+		PAST INFECTION
+	+			X		PAST INFECTION	+	-	X	PAST INFECTION
